# ERK1/2 Pathway-Mediated Differentiation of IGF-1-Transfected Spinal Cord-Derived Neural Stem Cells into Oligodendrocytes

**DOI:** 10.1371/journal.pone.0106038

**Published:** 2014-08-27

**Authors:** Bo Shi, Jianxun Ding, Yi Liu, Xinming Zhuang, Xiuli Zhuang, Xuesi Chen, Changfeng Fu

**Affiliations:** 1 Department of Spine Surgery, First Hospital of Jilin University, Changchun, P. R. China; 2 Key Laboratory of Polymer Ecomaterials, Changchun Institute of Applied Chemistry, Chinese Academy of Sciences, Changchun, P. R. China; Baylor College of Medicine, United States of America

## Abstract

Spinal cord injury (SCI) is a devastating event that causes substantial morbidity and mortality, for which no fully restorative treatments are available. Stem cells transplantation offers some promise in the restoration of neurological function but with limitations. Insulin-like growth factor 1 (IGF-1) is a well-appreciated neuroprotective factor that is involved with various aspects of neural cells. Herein, the IGF-1 gene was introduced into spinal cord-derived neural stem cells (NSCs) and expressed steadily. The IGF-1-transfected NSCs exhibited higher viability and were promoted to differentiate into oligodendrocytes. Moreover, the most possible underlying mechanism, through which IGF-1 exerted its neuroprotective effects, was investigated. The result revealed that the differentiation was mediated by the IGF-1 activated extracellular signal-regulated kinases 1 and 2 (ERK1/2) and its downstream pathway. These findings provide the evidence for revealing the therapeutic merits of IGF-1-modified NSCs for SCI.

## Introduction

According to the data from the National Spinal Cord Injury Statistical Center, the annual incidence of spinal cord injury (SCI) in the United States of America is estimated to be around 40 cases per million of population. SCI is a devastating neurological disorder that affected patients and their families, because it requires substantial long-term healthcare expenditure and permanently deprives of their life qualities [1]. Generally, SCI is highly heterogeneous, and the therapeutic approach differs depending on the location, extent, stage and time after the SCI. The current clinical therapeutic treatments for SCI mainly include surgical intervention, high doses of (MP) and symptomatic therapy followed by rehabilitation [2]–[4]. However, the above approaches all bear their own limitations. For example, surgery may lead to aggravating secondary damage by intraoperative blood loss and hypotension, and MP would increase the risk of infection, patients had a neurogenic bladder caused by spinal cord injury which can induce a strong resistance by long-term use of antibiotics [5]–[8]. Therefore, any novel therapeutic strategies for SCI that allow for major functional recovery would be a significant advance. The new techniques may provide the neuroprotective support for the remaining host cells, act as an anti-inflammatory treatment, and/or stimulate the regeneration of the adult central nervous system (CNS), and ultimately offer an effective therapy and improve the quality of the patient’s life.

With the rapid development of tissue engineering techniques targeting to the regeneration and repair of damaged tissue and organs, neural stem cells (NSCs) therapy has become a promisingly effective and novel treatment for the SCI patients. NSCs are present in the developing and also adult CNS, and can be isolated and expanded *in* *vitro* [9]. These multipotent cells can differentiate into neurons, oligodendrocytes, and astrocytes after transplantation, and subsequently promote the neural functional recovery [10]. However, NSCs can also differentiate into inappropriate cells, for example, resulting in tumor formation [11]. NSCs have a tendency to differentiate into glial cells after being transplanted into an impaired CNS. Moreover, NSCs could induce astrogliosis and the extension of a glial scar [12]–[14]. Therefore, how to build a microenvironment conducive to the survival and proper differentiation of NSCs is the key point for the application of NSCs to treat SCI.

Insulin-like growth factor 1 (IGF-1) is a 7.5 kDa polypeptide hormone. It has been shown to serve as a potent neurotrophic factor that promotes the growth of projection neurons, dendritic arborization, and synaptogenesis [15], [16]. Furthermore, it has been well established that exogenous IGF-1 could improve the transplant microenvironment and promote the survival of transplanted cells under different pathological conditions *in* *vitro* [17], [18]. These pieces of evidence lead us to hypothesize that the increased IGF-1 in NSCs would be beneficial for the cell survival and its subsequent differentiation into oligodendrocytes. Thus, in this study, we investigated the cell survival of IGF-1 expressing NSCs, the effect of IGF-1 on the cellular differentiation, and explored the possible mechanism underlying the neuroprotective effect of IGF-1.

## Materials and Methods

### Reagents

Cell culture medium, B27, fetal bovine serum, trypsin, and antibiotics were purchased from Gibco (Grand Island, NY, USA). Papain and DNase I were obtained from Sigma (St. Louis, Mo., USA). Lipofectamine 2000 was from Invitrogen (Grand Island, NY, USA). Antibodies against IGF-1, phosph-extracellular signal-regulated kinases 1 and 2 (phosph-ERK1/2), and β-actin were purchased from Santa Cruz Biotechnology (Santa Cruz, CA, USA), and anti-MBP was obtained from Abcam (Cambridge, England). Secondary antibodies were the products of Vector Lab (Burlingame, California, USA). Real-time polymerase chain reaction (RT-PCR) kit was purchased from Takara (Dalian, China). ERK inhibitor (*i.e.*, PD98059) was from Promega (Madison, Wisconsin, USA). Other chemicals were the products of Sigma.

### Expression Vectors

A pcDNA3.1 (+) plasmid backbone (Invitrogen, Carlsbad, CA) was used to construct the pcDNA3.1-IGF-1/GFP fusion gene expression vector. GFP gene was first PCR amplified from the template plasmid pEF-GFP by using the primers: GFP forward (Fw) 5′-ACTAGCGGCCGCATGGTGAGCAAGGGCGA-3′ and GFP reverse (Rev) 5′-ACTACTCGAGACAGCTCGTCCATGCCG-3′. The PCR product was digested with NotI and XhoI, and ligated to the pcDNA3.1 (+) after being digested with the same restriction enzymes. Then, rat IGF-1 gene was amplified from rat cDNA by using the primers Fw 5′-AAGCTTATGAGCGCACCTCCAATAAA3′ and Rev 5′-GGTACCCTACTTGTGTTCTTCAAGTGTACTTCC-3′, which were flanked by the restriction enzyme sites of HindIII and KpnI, respectively. The HindIII/KpnI double digested IGF-1 ORF was inserted into same restriction enzyme digested pcDNA-GFP. There were no mutations generated from the plasmid construction, and the IGF-1 gene remained in frame (data not shown). The vector contained the selectable neomycin resistance (NeoR) gene and an ampicillin resistance gene, which permitted the plasmid amplification in *Escherichia coli* DH5 alpha using standard amplification methods.

### Animal Procedure

The adult pregnant pathogen-free Wistar rats (300 to 350 g) were provided by the animal facility of Jilin University. All animals were handled under the protocol approved by the Institutional Animal Care and Use Committee of Jilin University, and all efforts were made to minimize suffering.

### Isolation and Culture of Spinal Cord-Derived NSCs

Spinal cords including cervical and thoracic regions were dissected from neonatal rats sacrificed by cervical dislocation as described previously [19]. In brief, spinal cord segments were washed 3 times in DMEM/F-12 medium, and the overlying meninges were removed. The dissected tissue was cut into ∼1 mm^3^ pieces, digested at 37°C for 30 min by 0.01% (w/v) papain and 0.01% (w/v) DNase I in DMEM/F-12 medium, and then mechanically triturated into a cell suspension. The cell suspension passed through a 40 µm Nylon mesh and centrifuged at 1,500 rpm for 10 min. Cells were resuspended in growth medium, which consisted of Neurobasal A medium supplemented with B27 neural supplement, 2 mM L-glutamine, 100 µg/ml penicillin-streptomycin, 5 µg/ml bovine insulin, 100 µg/ml human transferrin, 20 nM progesterone, 100 µM putrescine, 30 nM sodium selenite, 20 ng/ml human recombinant epidermal growth factor (EGF), and 20 ng/ml human recombinant fibroblast growth factor-2 (FGF2). Cells were seeded into petri dishes coated with matrigel at a density of 2×10^5^ cells/well and cultured at 37°C, 5% (v/v) CO_2_.

### Cell Transfection Procedure

Transfections were performed on the matrigel-coated 6-well plates using the Lipofectamine 2000 transfection reagent as described by the manufacturer. Transfected NSCs were kept for 3 h, and then the lipo-DNA complex was removed. Two days after transfection, 600 µg/ml of G418 was added to the growth medium, allowing the selective propagation of transfected cells in the culture. By day 14, the neo-resistant colonies appeared. Single transgenic colonies were isolated by a micropipette, dissociated into small clumps of cells and transferred into 4-well plates coated with matrigel. The cells proliferated continuously in the presence of 300 µg/ml of G418 and formed a large number of expanding colonies.

### Immunocytochemistry Analysis

Cultures were fixed with 4% (w/v) paraformaldehyde in 0.1 M phosphate-buffered saline (PBS) and washed with fresh PBS. Cultures were blocked with 10% (v/v) normal goat serum with 0.3% (v/v) Triton-X 100 and 1.5% (v/v) bovine serum albumin for 1 h at room temperature, and then incubated with the primary antibody of anti-MBP overnight at 4°C. Cultures were washed with 0.01 M PBS and then incubated with rabbit biotinylated secondary antibody (1∶200) for 1 h. After washes three times with PBS, the cells were incubated with DAB enhancing solution for 1 h at room temperature, and then detected by diaminobenzidine (DAB, sigma D5905). The staining was examined using a Nikon Eclipse TE 300 microscope.

### Western Blot Assays

For determination of ERK1/2 phosphorylation, cells were lysed and the total proteins were extracted by ice cold RIPA buffer containing 2 µg/ml leupeptin, 2 µg/ml aprotinin, and 100 µg/ml phenyl-methylsulphonyl fluoride. Protein concentrations were determined by the bicinchoninic acid (BCA) protein assay. Approximately 15 mg of whole cell lysate protein per lane was resolved by SDS-PAGE and transferred to a 0.22 µM polyvinylidene fluoride membrane. The nonspecific biding sites of membranes were blocked with bovine serum albumin for 1 h at room temperature. Membranes were incubated with rabbit anti-phospho ERK1/2 primary antibodies overnight at 4°C. β-actin served as an internal control. HRP-conjugated goat anti-rabbit IgG was used as the secondary antibody. Signals were detected by the enhanced chemiluminescence.

### RT-PCR Tests

Total RNA was isolated by Trizol reagent following the manufacturer’s instructions. RT-PCR was performed by One-Step PrimeScript RT-PCR Kit (Qiagen, Germany). The first strand cDNA was synthesized using 1.0 µg of total RNA and the oligo-dT as primers. The first strand cDNA serves as a template for the sequential PCR analysis. PCR conditions included denaturing at 94°C for 30 s, annealing at 55°C for 50 s, and extension at 72°C for 1 min, for 30 cycles. The detected genes and sequences of primers were listed in Table 1.

**Table 1:** Sequences of primers used for RT-PCR.

Gene	Fw primer	Rev primer
IGF-1	5′-GAGCGCACCTCCAATAAAGA-3′	5′-CTACATTCTTAGGTCTTGTTTCCT-3′
c*-myc*	5′-TACCCTCTCAACGACAGCAG-3′	5′-TCTTGACATTCTCCTCGGTG-3′
c*-fos*	5′-ATGATGTTCTCGGGTTTCAA-3′	5′-TGACATGGTCTTCACCACTC-3′
c*-jun*	5′-AGAGCGGTGCCTACGGCTACAGTAA-3′	5′-CGACGTGAGAAGGTCCGAGTTCTTG-3′
GAPDH	5′-ATGGAAGAAGAAATCGCCGC-3′	5′-ACACGCAGCTCGTTGTAGAA-3′

PCR products were separated by electrophoresis on a 2% (w/v) agarose gel containing ethidium bromide using a 100 bp DNA ladder for comparison. The expression of target genes and GADPH mRNA was indicated by measuring the density of the respective specific bands using the electrophoresis documentation and analysis. The amount of mRNA expression was determined by dividing the densitometry value of the mRNA RT-PCR product by that of the GAPDH product.

### Statistical Analysis

All experiments were independently done for at least three times. Data were expressed as mean ± standard deviation (SD). Results were evaluated by analysis of variance (ANOVA) using SPSS13.0 software. *p*<0.05 was considered to be statistically significant.

## Results

### Generation of IGF-1 expressing NSCs

**Figure 1:**
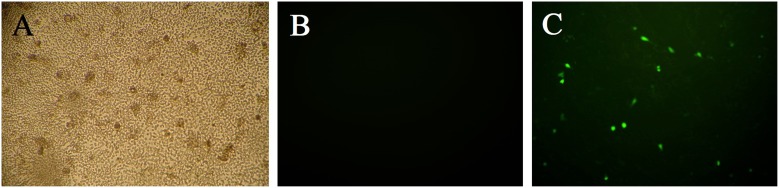
Stable IGF-1-GFP expression in spinal cord-derived NSCs. Phase-contrast image of pcDNA3.1-IGF-1/GFP-transfected NSCs selected by adding G418 for 14 days (A); fluorescence images of pcDNA3.1 (B) and pcDNA3.1-IGF-1-GFP transfected NSCs (C) at 24 h after transfection.

Spinal cord-derived NSCs were first obtained from new born rats. To generate stably IGF-1-expressed NSCs, the cells were transfected with cytomegalovirus (CMV) promoter-driven plasmid of pcDNA3.1-IGF-1/GFP. The plasmid contained a neomycin resistance cassette that allowed selection of successfully transfected NSCs by G418. At 48 h after transfection, G418 was added to select the GFP positive cells (Figure 1A), and the untransfected cells would not survive in the G418 containing medium. The optimal concentration of G418 for the specific selection was determined by applying with different concentrations of G418 over a 14-day period, the lowest effective concentration of 600 µg/ml, at which all the cells were killed off was chosen for selection. Fluorescent microscopy study showed that GFP-tagged IGF-1 was successfully expressed in the spinal cord-derived NSCs (Figure 1B and C), and the expression could occur at earlier 24 h after transfection.

**Figure 2:**
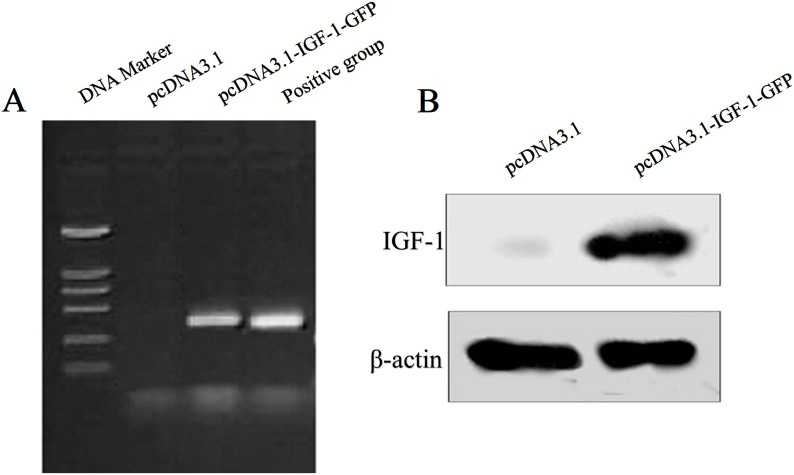
Confirmation of IGF-1/GFP expression in NSCs. RT-PCR analysis of IGF-1 in the pcDNA3.1 (lane 2) and pcDNA3.1-IGF-1/GFP-transfected NSCs (lane 3) (A). As shown in lane 1, DNA marker sizes were 100, 250, 500, 750, 1000 and 2000 bp from bottom to top. Similarly with the positive control (lane 4), the pcDNA3.1-IGF-1/GFP-transfected NSCs (lane 3) generate a 407 bp product that was identical with IGF-1 mRNA size. Western blot analysis of IGF-1 in the pcDNA3.1 (lane 1) and pcDNA3.1-IGF-1/GFP-transfected NSCs (lane 2) (B). IGF-1 protein can be detected in pcDNA3.1-IGF-1-GFP-transfected NSCs (lane 2) but not in the empty vector transfected cells (lane 1).

To further confirm that the transfected spinal cord-derived NSCs could express successfully the GFP-tagged IGF-1, IGF-1 mRNA level and its protein level were tested using RT-PCR and western blot, respectively. Figure 2A showed that IGF-1 mRNA was robustly expressed in the GFP-tagged IGF-1 construct transfected cells but not in the empty vector transfected cells. Similarly, the IGF-1 protein was also just blotted in the GFP-tagged IGF-1 construct transfected cells in comparison to the empty vector transfected cells (Figure 2B).

### Characterization of IGF-1 expressing spinal cord-derived NSCs

**Figure 3:**
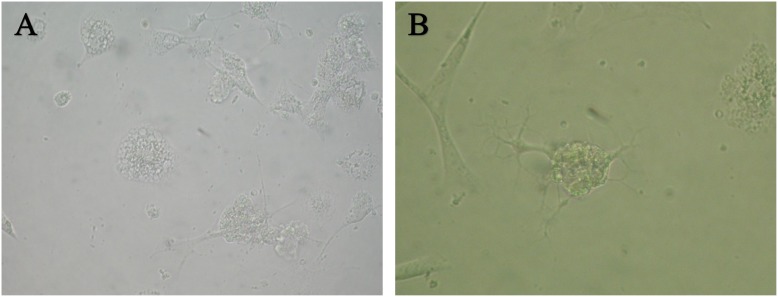
Morphological appearance of differentiated NSCs. Phase-contrast image of empty vector-transfected NSCs (A). Cells showed astrocyte-like morphology. Phase-contrast image of IGF-1-GFP expressing NSCs (B). Cells showed oligodendrocyte-like morphology.

**Figure 4:**
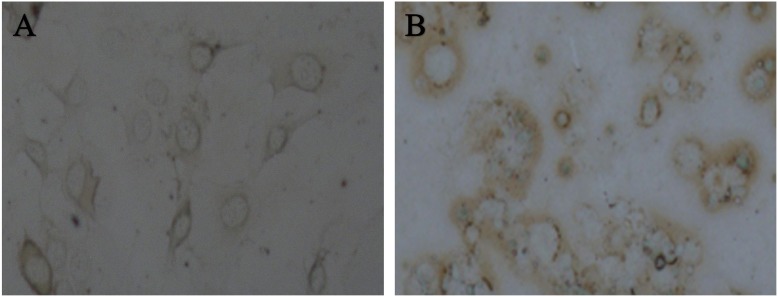
MBP expression in NSCs. Expression of MPB in NSCs transfected with pcDNA3.1 (A) and pcDNA3.1-IGF-1/GFP (B). As shown, MBP expression increased dramatically in the IGF-1-GFP expressing NSCs.

To evaluate the effect of IGF-1 expression on the spinal cord-derived NSCs, the cellular survival after introducing the pcDNA3.1-null plasmid or the pcDNA3.1-IGF-1/GFP plasmid into the cells were observed. As shown in Figure 3, in the pcDNA3.1-null group, more cells underwent apoptosis after transfection for 48 h (Figure 3A) and the survival cells developed protrusion then differentiated into astrocyte after transfection 7 to 10 days (Data not shown). In contrast, the pcDNA3.1-IGF-1/GFP transfected cells exhibited obviously well survive (Figure 3B). Moreover, a greater population of cells differentiated into oligodendrocytes. Furthermore, the MBP, a specific marker for oligodendrocyte, was examined by DAB staining. Compared with pcDNA3.1-null group (Figure 4A), the expression of MBP in IGF-1-transfected cells robustly increased (Figure 4B), indicating that IGF-1 could promote the differentiation of spinal cord-derived NSCs into oligodendrocytes.

### Effect of IGF-1 on the ERK1/2-related pathway

**Figure 5:**
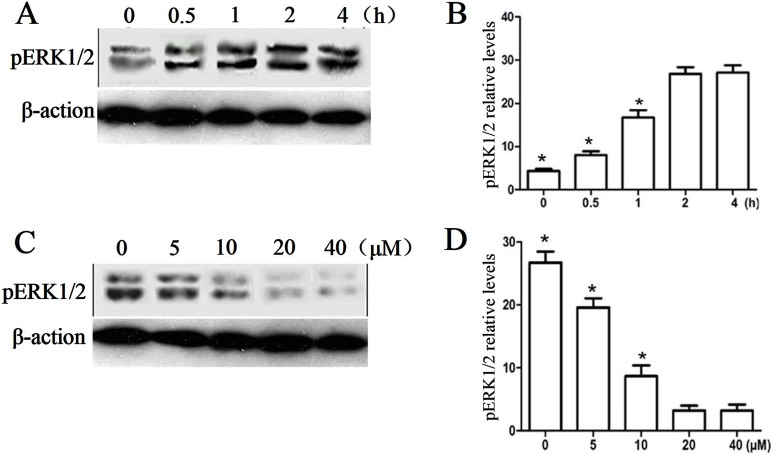
Effect of IGF-1 on the activation of ERK1/2. Western blot analysis of pERK1/2 in NSCs at different time points (0, 0.5, 1 or 2 h) after transfection of pcDNA3.1-IGF-1/GFP (A and B), and in IGF-1/GFP expressing NSCs treated with different concentrations of PD98059 (0, 5, 10, 20 or 40 µM) (C and D). *p<0.05 denotes as statistically significant in comparison to all other groups.

**Figure 6:**
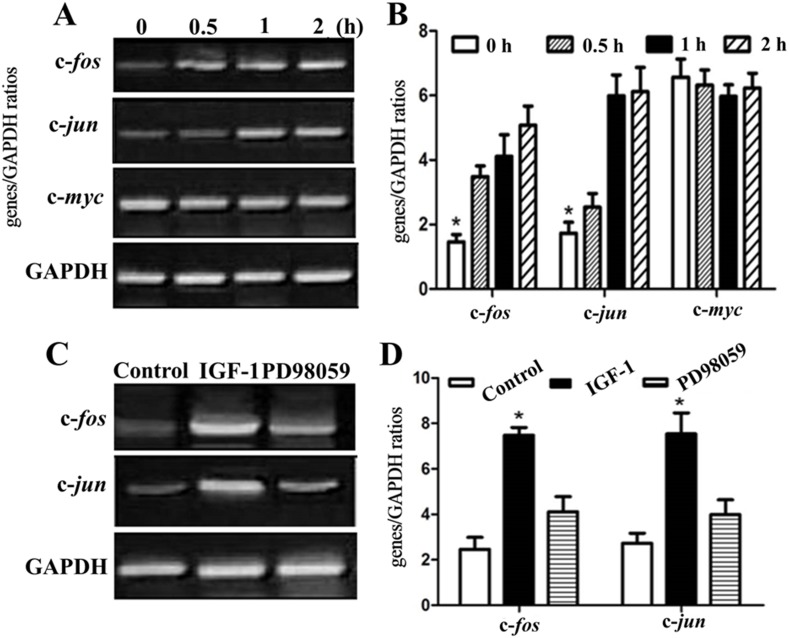
Effect of IGF-1 on the immediate genes of c*-fos,* c*-jun* and c*-myc* assessed by semi-quantitative RT-PCR analysis. Expressions of c*-fos*, c*-jun*, c*-myc* in IGF-1-transfected NCSs (A and B); c*-fos*, c*-jun* expression in NSCs were determined in control, or after IGF-1 expression and ERK1/2 inhibitor treatment (B and D). *p<0.05 denotes as statistically significant in comparison to all other groups.

Several lines of evidence have demonstrated that IGF-1 plays its neuroprotective role through ERK1/2 pathway. In order to investigate the mechanism underlying the IGF-1 neuroprotective effects for NSC therapy after SCI, phosphor-ERK1/2 (pERK1/2) was detected at several time points after the occurrence of IGF-1 expression. As shown in Figure 5A and 5C, with the increase of IGF-1 expression, the phosphorylation of ERK1/2 was dramatically enhanced over time, while the phosphorylation level reached a plateau till 4 h later, suggesting the activation of ERK1/2 pathway. Meanwhile, the specific ERK1/2 inhibitor (*i.e.*, PD98059) could markedly abolish the phosphorylation of ERK1/2 induced by IGF-1 (Figure 5B and 5D). Likewise, the downstream players including immediate genes of c-*fos*, c-*jun* and c-*myc* were also studied at 0, 0.5, 1 and 2 h after IGF-1 expression. The RT-PCR results (Figure 6A and 6C) showed that the mRNA level of c-*fos* and c-*jun* were significantly increased after the expression of IGF-1 at 0.5 and 1 h, while the c-*myc* mRNA had no change. And the specific inhibitor PD98059 could obviously inhibit the mRNA level increase of c-*fos* and c-*jun*, further suggesting that the phosphorylation of ERK1/2 and the subsequent activation of transcriptional factors of c-*fos* and c-*jun* mediated the neuroprotective effects of IGF-1.

## Discussion

The application of NSCs transplantation is highly restrained as the transplanted NSCs fail to positive differentiate, or mainly differentiated into glial cells [20]. It has demonstrated that the *in* *vivo* differentiation of NSCs is highly associated with the environmental cues [11]. Therefore, it would be promising to facilitate or promote the proper differentiation of transplanted NSCs through modifying the microenvironment in the lesion, such as by providing some trophic factors. In the present study, the IGF-1 was successfully introduced into NSCs and expressed steadily. The IGF-1-transfected NSCs can survive better and are promoted to differentiate into oligodendrocytes, which serve as a key contributor for the spinal cord to restore the function after injury. Moreover, we also found that the phenotype was induced through ERK1/2 pathway.

Insulin-like growth factor 1 (IGF-1), a single chain peptide of 70 amino acids, has been shown to influent the development and plasticity of CNS through promoting the growth of projection neurons, dendritic arborization, and synaptogenesis [15], [16], [21]. Accumulating evidence also illustrates that IGF-1 can have neuroprotective effects for neurons that are subject to a wide range of stressors. In addition, Yao *et al.* found that IGF-1 could reduce the lesion severity and clinical deficits in experimental autoimmune encephalomyelitis [22], [23]. Moreover, it has shown that a systematic single intravenous IGF-1 gene injection provides neuroprotective, anti-inflammatory, and anti-apoptotic effects in spinal cord hemi-sectioned rats [21]. Neuronal apoptosis is critically involved in the pathogenesis of SCI. Our results revealed that the IGF-1 expressing NSCs showed greater survival ability, which indicated that IGF-1 could inhibit the increase of apoptotic cells during the SCI due to its potent anti-apoptotic activity.

Morphological and physiological studies demonstrate that demyelination constitutes a significant component of the pathology of SCI, and then the recovery of conduction in demyelinated axons may permit to restore physiological function, which can be mediated by several mechanisms, such as remyelination by oligodendrocytes [24]. It has been reported that the oligodendrocyte stems possess an ability to initiate a restorative remyelination process after the exposure to some chemical or mechanical insults, resulting in the demyelination of axons within the adult CNS [25]. Some evidence have demonstrated that IGF-1 have effects on the oligodendrocyte biology during development [26]. IGF-1 can promote the long term survival of purified oligodendrocytes in culture [27]. Furthermore, IGF-1 can inhibit the mature oligodendrocytes apoptosis *in* *vitro* [28], [29]. Gene for myelin basic protein (MBP) is one of the genes that are activated during the differentiation of oligodendrocytes. In this work, IGF-1 could enable NSCs to survive better, and preferentially promote NSCs to differentiate into oligodendrocytes, which were indicated by the higher expression of MBP in IGF-1 expressing cells.

ERK1/2, the mitogen-activated protein (MAP) kinase family members, regulate a diverse array of cellular function [30]. The emerging evidences have indicated that ERK1/2 signalings are involved a variety of neural cellular processes including cell proliferation and survival, neoplastic transformation, neural plasticity and differentiation [31]–[33]. The pharmacological blockade of ERK1/2 activation leads to fewer oligodendrocytes with mature phenotypes, suggesting ERK1/2 pathway might play a vital role in oligodendrocyte differentiation [34], [35]. Moreover, Oh’s study implied that ERK1/2 could be a significant mediator for transmitting signals from the injury site to the cell body after contusive SCI, and further suggested that ERK1/2 activation might be involved in the enhanced outgrowth of corticospinal tract axons after treadmill training [36].

IGF-1 exerts its neuroprotective effects through the IGF-1 receptor that is linked to two major signaling pathways. and one of them is through the MAP kinase pathway. ERK1/2 is regulated by a cascade of phosphorylation including a dual phosphorylation at Thr/Tyr residues of the ERK1/2 activation domain that is carried out by MAP kinase kinases 1/2. Upon activation, the signal is relayed from the cell surface to the nucleus for targeting several transcription factors such as c-*jun* [37] and c-*fos* [38]. In our study, with the increasing expression of IGF-1 over time, the activation of ERK1/2 increased in parallel, and accordingly the c*-jun* and c*-fos* mRNA expression also increased, which can be abolished by the ERK1/2 specific inhibitor. It indicated that IGF-1 exerts its neuroprotective effects *via* ERK1/2-c-*fos*/c-*jun* pathway in NSCs.

## Conclusion

In summary, the continuous development of new strategies to treat SCI is urgently needed as there are no evident effective treatments for SCI till now. This study provides a strong evidence for studying the therapeutic merits of IGF-1 genetically modified NSCs for SCI. Indeed, more information is needed regarding genetic modification of NSCs, including transgene expression level and stabilization, elaborate gene regulation, and safety. A much more detailed characterization, namely of adult NSCs and their application in different injury models, severities, and treatment intervals will most likely increase the translation studies of these cells. Further experimental and clinical investigations will allow a better understanding of action mechanisms, therapeutic effects, and the safety profiles. In the near future, more molecules acting as overexpressing genes in NSCs and treating SCI will be recognized.
